# Effect of Copper (II) Sulfate on the Properties of Urea Formaldehyde Adhesive

**DOI:** 10.3390/polym14010094

**Published:** 2021-12-27

**Authors:** Hui Zhao, Xianzhen Li, Xi Wang, Mianwu Meng, Xiujian Wang, Siyu Huang, Weixing Gan

**Affiliations:** 1 Key Laboratory of Ecology of Rare and Endangered Species and Environmental Protection, Guangxi Normal University, Guilin 541004, China; zhaohui10042021@163.com (H.Z.); mmwmmw@mailbox.gxnu.edu.cn (M.M.); 2 College of Environment and Resources, Guangxi Normal University, Guilin 541004, China; lxzzb253983@163.com (X.L.); 90697@163.com (X.W.); 3 School of Chemistry and Pharmaceutical Sciences, Guangxi Normal University, Guilin 541004, China; wang1_xj@aliyun.com

**Keywords:** urea formaldehyde adhesive, free formaldehyde, formaldehyde release, reduction, copper (II) sulfate, mechanical properties

## Abstract

The purpose of this work is to investigate the effects of copper (II) sulfate on the formaldehyde release and the mechanical properties of urea formaldehyde (UF) adhesive. Copper (II) sulfate has been used as a formaldehyde scavenger in UF resin, and its effects on the physical and chemical properties of UF adhesive have been studied. Moreover, the mechanical properties and formaldehyde release of plywood prepared with modified UF resin have been determined. The UF resin has been characterized by Fourier-transform infrared (FTIR) spectroscopy and thermogravimetric analysis (TGA). FTIR spectra showed that the addition of copper (II) sulfate to the UF resin does not affect the IR absorptions of its functional groups, implying that the structure of UF is not modified. Further results showed that the free formaldehyde content of the UF resin incorporating 3% copper (II) sulfate was 0.13 wt.%, around 71% lower than that of the untreated control UF adhesive. With a copper (II) sulfate content of 3%, the formaldehyde release from treated plywood was 0.74 mg·L^−1^, around 50% lower than that from the control UF adhesive, and the bonding strength reached 1.73 MPa, around 43% higher than that of the control.

## 1. Introduction

Urea formaldehyde (UF) adhesive, phenolic adhesive, and melamine formaldehyde adhesive are the most widely used in wood-based panel production [1]. Of these, UF adhesive has the advantages of high bonding strength, fast curing, simple production, good corrosion resistance, low cost, and abundance of raw materials [2,3]. Consequently, there is a high demand for UF in the wood-based panel industry and in the wider adhesives market [4,5]. However, UF adhesive also has some disadvantages. For example, formaldehyde, one of the raw materials, is toxic to humans through inhalation, and harmful to the environment. Cured UF adhesive in wood-based panels can release formaldehyde into the environment [6]. Formaldehyde discharged into the environment causes both air and water pollution, which is especially significant when wood-based panels are used for indoor furniture and decoration [7,8]. To provide safety guidelines, the formaldehyde release of wood-based panels has been classified, for example, according to the Chinese national standard (GB 18580-2001), formaldehyde release levels are divided into E2 (≤5.0 mg·L^−1^) and E1 (≤1.5 mg·L^−1^) [9].

In order to avoid environmental pollution caused by high releases of formaldehyde and to protect the health of producers and consumers, it is necessary to improve the production process of UF adhesives, such that the amount of formaldehyde released is reduced, while maintaining the mechanical properties [10].

Both the presence of free formaldehyde in wood-based panels and the chemical decomposition of cured UF can give rise to formaldehyde release [11]. Free formaldehyde is the main release source during the initial use of an adhesive. At present, there are several methods for reducing formaldehyde release:

(1) Reducing the molar ratio of formaldehyde to urea (F/U). Low molar ratio UF resins generate less formaldehyde when they are used in wood-based products. However, this method has some disadvantages, such as a loss of bonding strength of the adhesive [12,13].
(2) Surface sealing method. Pibiri [14] showed that formaldehyde release can be reduced by 70% by applying a sealant coating to particleboard after 900 h. The advantage of such surface sealing is that it reduces the formaldehyde release during a certain period, but can only slow down the formaldehyde emission rate, the overall formaldehyde content in the board remains the same.
(3) Using a formaldehyde adsorbent or scavenger. Inorganic formaldehyde adsorbents, such as alumina [15], attapulgite [16], and melamine [17], can physically adsorb formaldehyde and retard its release, but they do not change the total amount of formaldehyde within the material. Organic formaldehyde scavengers, such as tannin [18], montmorillonite [19], and methylamine, or ethylamine [10], react chemically with free formaldehyde, thereby reducing its content in an adhesive, and hence its release from UF products. A study by Ghani et al. [10] revealed that the addition of 1% propylamine to UF resin reduced the formaldehyde release from particleboard by 56%, but it also had a detrimental effect on the mechanical properties of the product.

In order to manufacture UF resin characterized by a low formaldehyde release and good mechanical properties, new methods or processes must be adopted. In this study, we have used copper sulfate as an additive, and have studied its effect on the properties of UF adhesive.

Formaldehyde has a strong reducing ability. It is often used as a reducing agent in electroless copper plating [20] to reduce Cu^2+^. Hence, Cu^2+^ may oxidize formaldehyde to much less toxic HCOO^−^, to reduce the amount of free formaldehyde in UF adhesive. Copper (II) sulfate was once used as an antibacterial agent in UF resin [21,22]. In this work, UF resin with an F/U molar ratio of 1.4 has been synthesized. Copper (II) sulfate has been used as a formaldehyde oxidant, and its effect on the free formaldehyde content in UF adhesive has been studied. The UF adhesive modified with copper (II) sulfate has been applied to plywood. The mechanical properties and formaldehyde release of the prepared plywood have been studied and analyzed. Attenuated total reflectance Fourier-transform infrared (ATR-IR) spectroscopy and thermogravimetry have been applied to delineate the role of copper (II) sulfate in UF resin.

## 2. Materials and Methods

### 2.1. Materials

Copper (II) sulfate (AR, Guangdong Guanghua Technology Co., Ltd., Guangzhou, China), melamine, ammonium chloride (AR, Sinopharm Chemical Reagent Co., Ltd., Shanghai, China), formic acid, sodium hydroxide, urea, formaldehyde solution (AR, Xilong Science Co., Ltd., Shantou, China), and food grade flour were obtained commercially. Eucalyptus veneer (moisture content 8–12%, 480 mm × 480 mm × 1.7 mm) was procured from a lumber mill.

### 2.2. Equipment

The equipment used comprised a flat vulcanizer (XLB-D, Huzhou Shunli Rubber Machinery Co., Ltd., Huzhou, China) and a universal mechanical testing machine (MWW-10A, specification 10 kN, Jinan Xinguang Testing Machine Manufacturing Co., Ltd., Jinan, China). IR spectra were obtained on a Fourier-transform infrared spectrometer (Spectrum One System, Perkin-Elmer, Waltham, MA, USA), and thermograms were obtained on a synchronous thermal analyzer (STA-449C, Netzsch, Selb, Germany).

### 2.3. Experimental Methods

#### 2.3.1. Preparation of UF Resin Adhesive

37% formaldehyde solution (454 g) was placed in a 1000 mL 4-necked flask immersed in a water bath. Under stirring, urea (240 g) was added in 3 portions (to give a final F/U molar ratio of 1.4). (1) Urea (156 g) was added as the first batch, and the mixture was adjusted to pH 8–9 with 40% aqueous NaOH solution. (2) The water bath was heated to 88 °C, whereupon the second batch of urea (60 g) was added. After 20 min at 88 °C, the mixture was adjusted to pH 5.0–5.5 with 40% HCOOH solution, and the reaction was allowed to proceed until the solution reached the desired viscosity. (3) The mixture was then adjusted to pH 7.5–8.0 with 40% aqueous NaOH solution, and the reaction solution was allowed to cool to 60 °C, whereupon the final portion of urea (24 g) and melamine (7 g) were added. The solution was kept at 60 °C for 15 min, and then allowed to cool to room temperature to obtain UF resin (A).

#### 2.3.2. Preparation of Copper (II) Sulfate/UF Resin Adhesive

A certain proportion of copper (II) sulfate was added to the requisite amount of synthetic UF resin adhesive (A), and the mixture was mechanically stirred at 60 °C for 1 h to obtain copper (II) sulfate/UF resin adhesive (B).

#### 2.3.3. Preparation of Plywood

NH_4_Cl (0.66 g) and flour (13 g) were added to UF resin (66 g) and the mixture was stirred to obtain the final adhesive (C). Adhesive C was coated on either side of a eucalyptus veneer using a rubber scraper. Three layers of plywood were assembled. They were pre-pressed for 20 min, and then hot-pressed at 110–120 °C and 1.2 MPa for 300 s [23,24].

### 2.4. Determination of Free Formaldehyde in the UF Resin

The content of free formaldehyde in the UF resin adhesive was determined by NaOH titration [10].

### 2.5. Performance Tests of Plywood

#### 2.5.1. Bonding Strength Test of Plywood

Plywood samples were prepared according to the Chinese national standard GB/T9846-2015 [25]. Bonding strengths were tested according to the Chinese national standard GB/T17657-2013 [26].

#### 2.5.2. Test of Formaldehyde Release from Plywood

According to the Chinese national standard GB/T17657-2013, the formaldehyde release from plywood was tested by the dryer method [26].

### 2.6. FTIR Characterization of UF Resin

The synthesized UF resin was freeze-dried (−70 °C) and ground to a powder.

The ATR-IR spectrum of UF sample E was acquired on the abovementioned PerkinElmer Spectrum One system in the wavelength range 4000–400 cm^−1^ with a resolution of 4 cm^−1^.

### 2.7. TG Analysis of UF Resin

UF sample E was ground to a powder, and a sample of 3–10 mg was used for TGA on the abovementioned STA-449C thermal analyzer. The temperature range was 30–600 °C, the heating rate was 10 °C·min^−1^, and the protective gas was nitrogen.

## 3. Results and Discussion

### 3.1. Effects of Copper (II) Sulfate on the Properties of UF Resin Adhesive

**Figure 1:**
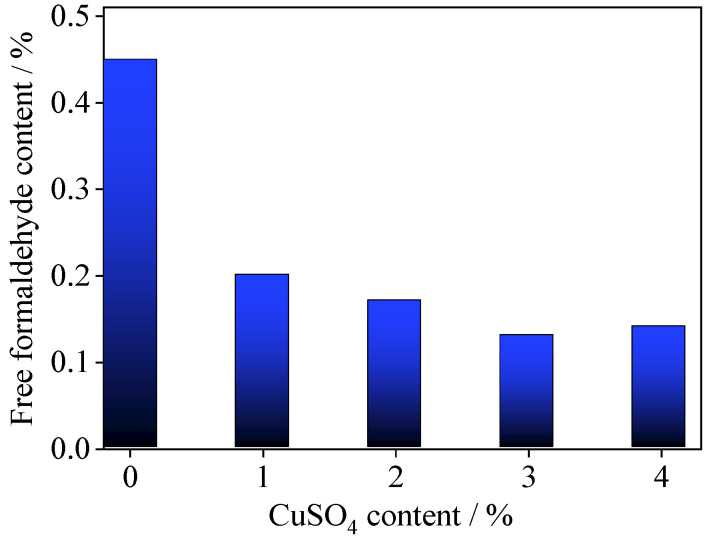
Relationship between copper (II) sulfate content in UF resin adhesive and the free formaldehyde content.

Figure 1 shows the effect of the amount of copper (II) sulfate on the free formaldehyde content of the UF adhesive. The content of free formaldehyde in the control UF adhesive was 0.45%, and it decreased with increasing copper (II) sulfate content. This was due to the oxidation of free formaldehyde by Cu^2+^ ions to form formate [27,28]. When 1% copper (II) sulfate was added to UF resin adhesive, the content of free formaldehyde decreased by 55%, surpassing the decrease (50%) achieved with an amine scavenger [10]. When 3% copper (II) sulfate was added to the UF resin adhesive (UF/3%CuSO_4_), the free formaldehyde content therein was 0.13 %, around 71% lower than that of the control UF adhesive. This free formaldehyde content is considerably lower than the value of 0.3% in UF adhesive for plywood stipulated by Chinese standard GB/T 14732-2017 [29].

### 3.2. Effect of Copper (II) Sulfate Content on the Properties of Plywood

Plywood was prepared with UF resin adhesive, and the effects of copper (II) sulfate on its bonding strength and formaldehyde release were tested.

#### 3.2.1. Effect of Copper (II) Sulfate Content on the Bonding Strength of Plywood

**Figure 2:**
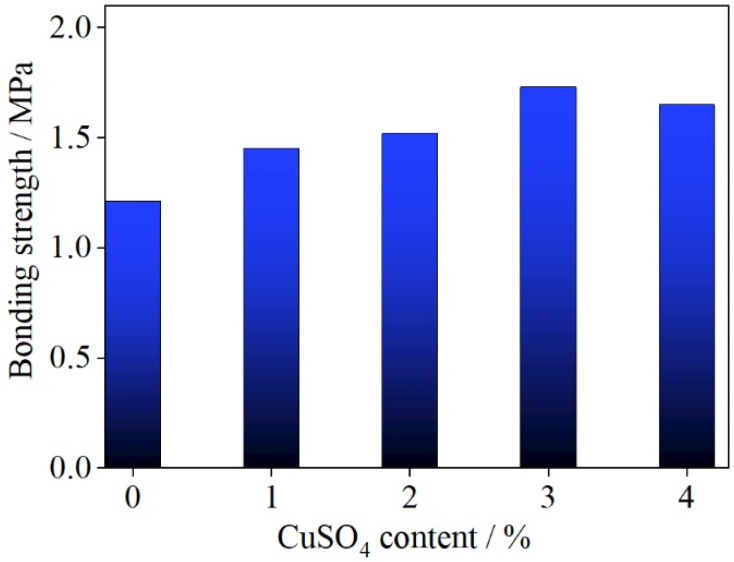
Relationship between copper (II) sulfate content in UF resin adhesive and the bonding strength of plywood.

Figure 2 shows the relationship between copper (II) sulfate content in UF resin adhesive and the bonding strength of plywood. It can be seen from Figure 2 that the bonding strength of the plywood prepared without copper (II) sulfate adhesive was 1.21 MPa, and that this parameter increased with increasing copper (II) sulfate content in the range 0–3%. This is at variance with previously reported results, which implied that the content of copper (II) sulfate has little effect on bonding strength [22]. Our finding can be attributed to the redox reaction of copper (II) sulfate and formaldehyde generating small particles of Cu or Cu_2_O. According to the research of Mahrdt et al. [30], the incorporation of small particles into UF resin can help to overcome the limitation of polymer composition, increase the coverage of the adhesive on a wood surface, and improve the strength of the adhesive. When the copper (II) sulfate content was 3%, the bonding strength reached 1.73 MPa, around 43% higher than that of the control UF adhesive. Ghani et al. [31] reported that the adhesion strength decreased significantly with the addition of an amine as a formaldehyde scavenger. The observed deterioration in the mechanical properties was attributed to a decrease in the degree of crosslinking in the curing network [32] caused by the reduction of free formaldehyde content as a result of its reaction with the amine additive.

Thus, the addition of copper (II) sulfate not only reduces the free formaldehyde content of UF resin adhesive, but also improves the bonding strength of plywood, which is very different from the action of an amine formaldehyde scavenger.

#### 3.2.2. Effect of Copper (II) Sulfate Content on Formaldehyde Release from Plywood

**Figure 3:**
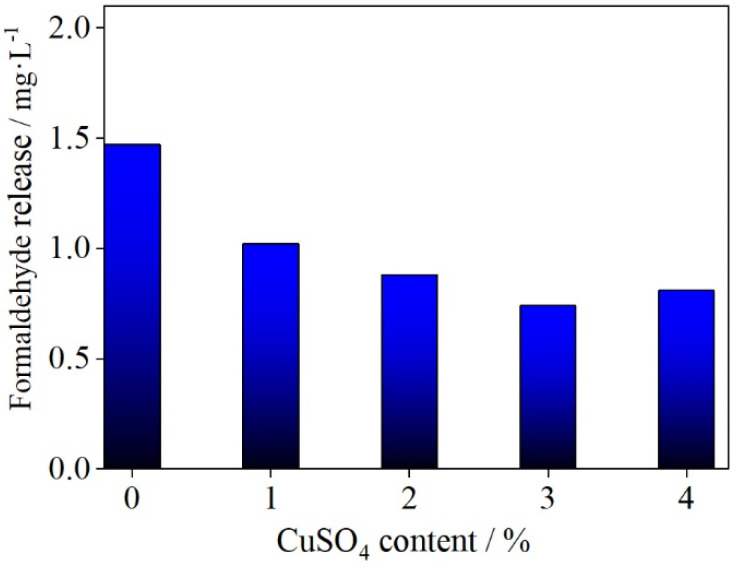
Relationship between copper (II) sulfate content in UF resin adhesive and formaldehyde release from plywood.

Figure 3 shows the relationship between copper (II) sulfate content in UF resin adhesive and formaldehyde release from plywood. It can be seen from Figure 3 that the formaldehyde release from plywood prepared with adhesive without copper (II) sulfate was 1.47 mg·L^−1^, within the Chinese national standard (GB 18580-2001) level E1 (≤1.5 mg·L^−1^) [9], but very much at the high end of this range. Formaldehyde release from plywood decreased with increasing copper (II) sulfate content in the range 0–3%. When the copper (II) sulfate content was 3%, the formaldehyde release from plywood was 0.74 mg·L^−1^, around 50% lower than that from the control UF adhesive, and well within the Chinese national standard E1 formaldehyde release standard range.

Bonding strength and formaldehyde release are two important performance parameters in urea resin adhesives, but they are difficult to mutually reconcile. The bonding strength is highly dependent on the F/U molar ratio in UF resin [13]. The higher the F/U molar ratio, the better the bonding strength, but formaldehyde release will inevitably increase accordingly. In order to prepare UF resin with a low formaldehyde release to meet environmental requirements, a lower F/U molar ratio is generally used. Consequently, some performance, such as bonding strength, is sacrificed. For example, a UF resin with an F/U molar ratio of only 0.8 was used by Park et al. [33]. A formaldehyde scavenger can be added to UF resin adhesive to reduce formaldehyde release. De Cademartori et al. [15] added 2 wt.% Al_2_O_3_ nanoparticles as a formaldehyde scavenger to reduce formaldehyde release from a resin, but the reduction achieved amounted to just 14%. A 7% propylamine loading, as a formaldehyde scavenger in UF resin, reduced the formaldehyde release by 52%, but a 60% loss in mechanical properties was incurred [31].

Here, copper (II) sulfate as a formaldehyde remover was used in UF resin with an F/U molar ratio of 1.4. It reduced both the free formaldehyde content and the formaldehyde release, while also improving the bonding strength of the adhesive. When the UF resin contained 3% copper (II) sulfate, its overall performance was better. The free formaldehyde content was 0.13%, around 71% lower than that of the control UF adhesive; the formaldehyde release from plywood was 0.74 mg·L^−1^, around 50% lower than that with the control UF adhesive; and the bonding strength of plywood reached 1.73 MPa, around 43% higher than that with the control UF adhesive.

### 3.3. ATR-IR Characterization of the UF Resin

**Figure 4:**
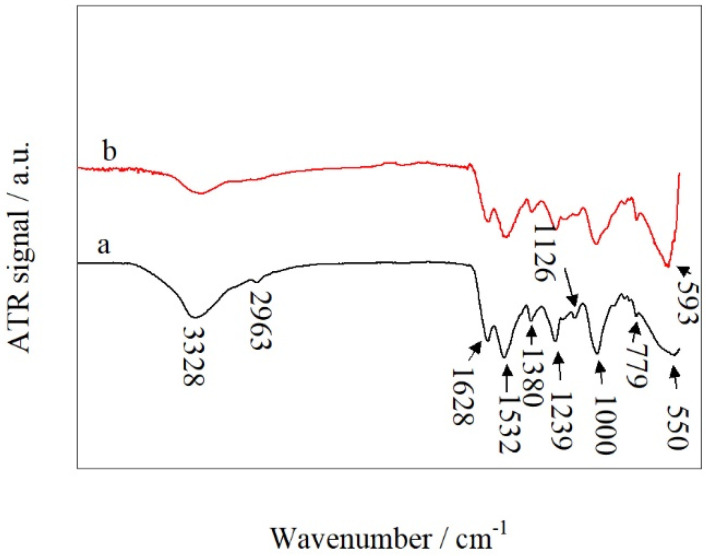
ATR-IR spectra of (**a**) the control UF and (**b**) UF/3% CuSO_4_.

Figure 4 shows ATR-IR spectra of UF resin before and after the addition of copper (II) sulfate. The ATR-IR spectrum of the control UF resin (Figure 4a) remained largely unchanged by the addition of 3% copper (II) sulfate (Figure 4b). The broad IR absorption peak at 3328 cm^−1^ can be attributed to the stretching vibrations of O–H and N–H bonds [34]. The peak at 2960 cm^−1^ can be attributed to asymmetric C–H stretching vibrations in –CH_2_– [35]. The peak at 1628 cm^−1^ can be attributed to the C=O stretching vibration in the saturated aliphatic aldehyde group [36]. The peak at 1380 cm^−1^ can be attributed to a C–N bending vibration, and a further peak at 1240 cm^−1^ can be attributed to a C–O stretching vibration [37]. Three further IR absorption peaks at 1126, 1000, and 779 cm^−1^ may be attributed to the stretching vibrations of C–O–O and C–O–H and the in-plane rocking vibration of –CH_2_–, respectively [2].

The ATR-IR spectrum of the control UF resin (Figure 4a) features a peak at 550 cm^−1^. This peak is shifted to 593 cm^−1^, following the addition of 3% copper (II) sulfate (Figure 4b). This shift can be attributed to the presence of Cu_2_O formed by the reaction of copper (II) sulfate and formaldehyde in the solution [38].

### 3.4. Thermogravimetric Analysis of UF Resin

**Figure 5:**
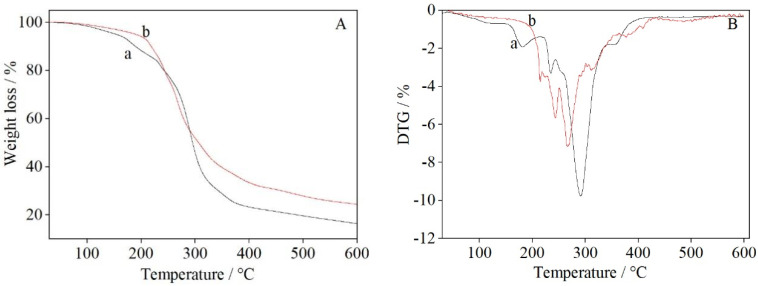
TG (**A**) and DTG (**B**) traces of (**a**) the control UF, and (**b**) UF/3% CuSO_4_.

Figure 5 shows the thermogravimetric (TG; A) and differential thermogravimetric (DTG; B) traces of the UF resin. It can be seen from the TG traces in Figure 5A that the thermal degradation processes of UF resin and UF/3% CuSO_4_ resin can be mainly divided into three stages [39].

(1) The first process is the evaporation of residual water and the release of formaldehyde from the UF resin at 30–200 °C [39], which includes water and formaldehyde produced by the condensation reaction of hydroxymethyl and amine groups at high temperatures. The mass loss is about 12% (see Figure 5A(a)). Figure 5A(b) shows that copper (II) sulfate had a significant effect on the thermal behavior of the UF resin, with the mass loss from the UF/3% CuSO_4_ resin being only about 7% at 200 °C. The decrease in mass loss at 200 °C can be related to the low content of free formaldehyde in the modified UF resin, as well as the interaction between water and Cu_2_O, the reduction product of copper (II) sulfate, through hydrogen bonding [40]. It can be seen from the DTG traces in Figure 5B that UF resin displayed a small peak in the range 90–150 °C and a peak in the range 150–200 °C, whereas UF/3% CuSO_4_ resin showed no obvious peak in the range 90–200 °C. This indicated that the release of water and free formaldehyde from the UF/3% CuSO_4_ resin was significantly suppressed.
(2) The second stage involves mass losses caused by the breaking and decomposition of the methylene ether, and methylene bonds, and the removal of other small molecules from the resin at 200–350 °C [41]. The cumulative weight losses from UF resin and UF/3% CuSO_4_ resin in this region were about 77% and 66%, respectively.
(3) The third mass loss stage occurs in the range 350–600 °C [41]. In this stage, volatile elements are removed, and the resin is carbonized. The cumulative weight losses from the UF and UF/3% CuSO_4_ resins at 600 °C were 83% and 75%, respectively. The lower mass loss from the UF resin incorporating CuSO_4_ may be attributed to the shielding effect caused by the presence of metal oxide particles or a greater residual mass, and does not necessarily represent an increase in thermal stability [15].

## 4. Conclusions

In the present paper, the effects of copper (II) sulfate on the properties of UF resin have been studied. The results showed that the content of free formaldehyde decreased with the increase in copper (II) sulfate content in the range 0–3 wt.%. It was reduced to 0.13% by the incorporation of 3% copper (II) sulfate, about 71% lower than that of the control UF adhesive.

Thermogravimetric analysis showed that UF resin incorporating copper (II) sulfate underwent lower formaldehyde and water release than the control UF resin in the range 20–100 °C. FTIR spectra have shown that the addition of copper (II) sulfate does not affect the IR absorptions of functional groups in the UF resin, and hence does not affect its structure, except for a new absorption peak attributable to Cu_2_O formed by the reaction of Cu^2+^ with free formaldehyde in the resin. In analyzing the performances of plywood samples, the bonding strength was increased with increasing copper (II) sulfate content in the range 0–3 wt.%, while formaldehyde release was decreased. The bonding strength of UF/3% CuSO_4_ resin was about 43% higher than that of the control. UF resin and its formaldehyde release was about 50% lower.

## Data Availability

The data presented in this study are available on request from the corresponding author.
